# Outcome and prognostic factors in critically ill patients with systemic lupus erythematosus: a retrospective study

**DOI:** 10.1186/cc3481

**Published:** 2005-02-25

**Authors:** Chia-Lin Hsu, Kuan-Yu Chen, Pu-Sheng Yeh, Yeong-Long Hsu, Hou-Tai Chang, Wen-Yi Shau, Chia-Li Yu, Pan-Chyr Yang

**Affiliations:** 1 Division of Pulmonary Medicine, Department of Internal Medicine, National Taiwan University Hospital, Taipei, Taiwan; 2 Assistant Professor, Graduate Institute of Clinical Medicine, College of Medicine, National Taiwan University, Taipei, Taiwan; 3 Professor, Division of Rheumatology, Department of Internal Medicine, National Taiwan University Hospital, Taipei, Taiwan; 4 Professor, Division of Pulmonary Medicine, Department of Internal Medicine, National Taiwan University Hospital, Taipei, Taiwan

## Abstract

**Introduction:**

Systemic lupus erythematosus (SLE) is an archetypal autoimmune disease, involving multiple organ systems with varying course and prognosis. However, there is a paucity of clinical data regarding prognostic factors in SLE patients admitted to the intensive care unit (ICU).

**Methods:**

From January 1992 to December 2000, all patients admitted to the ICU with a diagnosis of SLE were included. Patients were excluded if the diagnosis of SLE was established at or after ICU admission. A multivariate logistic regression model was applied using Acute Physiology and Chronic Health Evaluation II scores and variables that were at least moderately associated (*P* < 0.2) with survival in the univariate analysis.

**Results:**

A total of 51 patients meeting the criteria were included. The mortality rate was 47%. The most common cause of admission was pneumonia with acute respiratory distress syndrome. Multivariate logistic regression analysis showed that intracranial haemorrhage occurring while the patient was in the ICU (relative risk = 18.68), complicating gastrointestinal bleeding (relative risk = 6.97) and concurrent septic shock (relative risk = 77.06) were associated with greater risk of dying, whereas causes of ICU admission and Acute Physiology and Chronic Health Evaluation II score were not significantly associated with death.

**Conclusion:**

The mortality rate in critically ill SLE patients was high. Gastrointestinal bleeding, intracranial haemorrhage and septic shock were significant prognostic factors in SLE patients admitted to the ICU.

## Introduction

Systemic lupus erythematosus (SLE) is an archetypal autoimmune disease, involving multiple organ systems and with varying course and prognosis. Even though the survival rate among SLE patients has improved over the past few decades [1-3], there remain a host of factors that are associated with death in SLE patients, including the level of disease activity and demonstrable organ damage at presentation [4,5]. Moreover, coronary artery disease has increasingly been recognized to be an important cause of death in SLE patients [6]. In contrast, infections, which develop in the setting of active SLE under aggressive treatment, are often difficult to identify as a single cause of death [7]. Effective treatment for SLE has led to improved prognosis and extended survival times [8,9]. However, intensive treatment concomitantly results in an increased number of disease- or therapy-associated complications, which also require intensive care. Patients with SLE admitted to the intensive care unit (ICU) mostly present with florid disease manifestations, with a compendium of pathologies precipitating the admissions [10]. However, there is a paucity of clinical data regarding prognostic factors in SLE patients admitted for intensive care.

In the present study we analyzed prognostic factors in a cohort of SLE patients admitted to our ICU over the past 8 years, particularly with respect to causes of ICU admission, severity of illness and clinical course during the patients' ICU stays.

## Materials and methods

### Patients

All patients with SLE admitted to the medical ICU of the National Taiwan University Hospital from January 1992 to December 2000 were included. Diagnosis of SLE was confirmed if the patient fulfilled at least four of the 1982 American Rheumatism Association revised classification criteria [11]. The exclusion criterion was diagnosis of SLE at or after admission to the ICU. If the patient was admitted to the ICU more than once, only data from the first ICU admission were analyzed.

### Data collection

We analyzed the following clinical and laboratory parameters: age, sex, underlying diseases and associated manifestations of SLE, causes of admission, Acute Physiology and Chronic Health Evaluation (APACHE) II score [12], arterial oxygen tension/inspired fractional oxygen ratio, complete blood count, characteristics of lesions on chest radiographs, sites of infection and organisms cultured, treatments administered during the patient's ICU stay, occurrence of complications, duration of ICU study and outcome.

The cause of ICU admission was defined as the major problem necessitating admission to the ICU. This was determined on the basis of clinical data. Cardiogenic pulmonary oedema is due to poor cardiac performance. Noncardiogenic pulmonary oedema is due to fluid overloading of a noncardiogenic cause. APACHE II scores were calculated using clinical data available from the first 24 hours of intensive care. The median APACHE II score was used as a cutpoint to classify the patients into high or low score groups. Renal involvement was defined as urinary excretion of more than 500 mg protein/24 hours, cellular casts not attributable to infection, or abnormal histology on renal biopsy. Abnormal complete blood count was defined as haemolytic anaemia or leucopenia (<4 × 10^9^/l), lymphopenia (<1.5 × 10^9^/l), or thrombocytopenia (<100 × 10^9^/l) in the absence of offending drugs. Neutropenia was defined as an absolute neutrophil count under 1.0 × 10^9^/l. Pneumonia was defined as new and persistent radiographic opacity, positive sputum culture and any three of the following: body temperature above 38°C, white blood cell count above 15 × 10^9^/l, increased airway secretions, or worsening gas exchange [13]. Respiratory failure was defined as arterial oxygen tension below 60 mmHg and/or arterial carbon dioxide tension of 50 mmHg or greater while the patient was breathing room air. Acute respiratory distress syndrome (ARDS) was defined in accordance with to the American–European Consensus Conference on ARDS [14]. Sepsis and septic shock were defined in accordance with the criteria of Bone and coworkers [15].

Gastrointestinal bleeding was defined as the presence of at least one of the following: melena, haematemesis, or blood from nasogastric aspirate over 24 hours. Finally, patient outcome was classed as death while the patient was in the ICU or survival to discharge from the ICU.

### Statistical analysis

Values are expressed as median (range) for continuous variables, or as a percentage of the group from which they were derived for categorical variables. Differences in survival among subgroups of variables were analyzed by χ^2^ test or by Fisher's exact test when necessary. A forward stepwise multivariate logistic regression model was applied (SPSS 10.0 for Windows; SPSS Inc., Chicago, IL, USA), using APACHE II score and variables that were at least moderately associated (*P* < 0.2) with survival in the univariate analysis. *P* ≤ 0.05 was considered statistically significant.

## Results

### Clinical characteristics

From January 1992 to December 2000, a total of 4235 patients were admitted to the ICU. Of these, 51 SLE patients were included in the present study. The clinical features of the 51 SLE patients are summarized in Table 1. Three of the 51 patients had associated autoimmune disease in addition to SLE, including one with polymyositis, one with Graves' disease and one with psoriasis. The most common disease manifestation among the 51 SLE patients before ICU admission was mucocutaneous involvement (44 [86.2%]), followed by renal involvement (37 [72.5%]). The median duration from diagnosis of SLE to ICU admission was 27 months (range 1–288 months). Forty-seven patients (92.2%) were receiving corticosteroid medication before ICU admission, with a mean equivalent dose of 20 mg/day prednisolone.

### Causes of admission

A total of 60 ICU admissions were included in the present study, with the annual number of admissions of SLE patients fluctuating. No trend favouring any particular cause of ICU admission was identified during the course of the study. There were seven patients with more than one admission to the ICU, including five patients with two admissions and two with three admissions. The causes of ICU admission are summarized in Table 2. The most common cause of admission to the ICU was pneumonia with ARDS (14 [23%]).

#### Noninfectious causes

Thirty-three (55.0%) admissions to the ICU were due to noninfectious problems. For patients in the cardiogenic category, heart failure was the major cause of admission, including cardiogenic shock and cardiogenic pulmonary oedema. Nine (15.0%) admissions were for pericardial effusion. Among them, three patients were admitted because of cardiac tamponade. Eleven patients had noninfectious pulmonary problems, and noncardiogenic pulmonary oedema was the most common cause. Among the patients with noncardiogenic pulmonary oedema, all were due to acute deterioration in renal function. For patients in the neurological category, status epilepticus was the most common cause of admission, and most (71.4%) had a previous history of seizures.

#### Infectious causes

Twenty-seven admissions (45.0%) to the ICU were due to infectious diseases, including pneumonia with ARDS and sepsis of extrapulmonary origin (Table 3). The infectious pathogens identified in SLE patients varied considerably. Eleven had positive blood culture results, including six Gram-negative bacilli, four Gram-positive cocci and one fungaemia. *Pseudomonas aeruginosa* (*n* = 3), *Salmonella* (*n* = 2; groups B and C) and *Escherichia coli* (*n* = 1) accounted for the cases of Gram-negative sepsis, whereas *Staphylococcus aureus* (*n* = 2; including one methicillin-resistant *S aureus*), *Staphylococcus epidermidis* (*n* = 1) and *Streptococcus pneumoniae* (*n* = 1) were the major pathogens of Gram-positive sepsis. Three patients had confirmed positive pleural effusion culture, including one methicillin-resistant *S aureus*, one *S pneumoniae* and one *Acinetobacter baumannii*. One patient suffered from disseminated tuberculosis with tuberculous bacilli isolated from pleural effusion and ascites. One patient had tuberculous meningitis, with tuberculous bacilli isolated from the cerebrospinal fluid.

### Clinical course, treatment and outcome

The clinical courses and outcomes in the 51 patients for their first admissions are summarized in Table 3. In order to assess the possible effect of repeat measurement, the results were analyzed separately by all admissions and first admission only; no significant differences were noted.

Forty-one patients were receiving steroid therapy to control the activity of the disease, including seven receiving pulse therapy (equivalent dose of >625 mg/day prednisolone). Also, 35 patients required mechanical ventilation, with three undergoing tracheotomy because of prolonged intubation. Nineteen patients needed dialysis, including 11 who received continuous venovenous haemofiltration because of unstable haemodynamics.

Fifteen (29.4%) had gastrointestinal bleeding during their ICU stay, which manifested as melena, haematemesis, or blood in the nasogastric aspirate. The rate of steroid use was higher in patients with gastrointestinal bleeding than in those who had no gastrointestinal bleeding (87.5% versus 75%), but the association was not statistically significant (*P* = 0.253). No evidence of mesenteric vasculitis could be demonstrated in the patients with gastrointestinal bleeding. One of them had colon perforation and underwent surgical intervention, whereas in the others the bleeding was controlled by medication without the need for fluid resuscitation or blood component therapy. Four developed pneumothorax during their ICU stay and were treated by tube thoracotomy for drainage.

Intracranial haemorrhage occurred in six patients (11.7%), including four with brainstem haemorrhage, one with subarachnoid haemorrhage and one with frontal lobe haemorrhage. Three patients were admitted to the ICU because of intracranial haemorrhage; these were not included in the six patients.

Whereas the overall mortality of the non-SLE ICU population was 29.0% from 1992 to 2000, the mortality rate for SLE patients admitted to the ICU was 47.0%.

### Prognostic factors

To identify prognostic factors for death in SLE patients admitted to the ICU, univariate and multivariate analyses for these factors were conducted. We performed the analyses using data from the first admission of the patients. Table 4 summarizes the variables with at least moderate influence (*P* < 0.2) on mortality, as determined by univariate analysis. Patients with abnormal complete blood count on admission (*P* = 0.005), with intracranial haemorrhage occurring while in the ICU (*P* = 0.018), with complicating gastrointestinal bleeding in the ICU (*P* = 0.01), and with concurrent septic shock in the ICU (*P* < 0.001) were at higher risk of mortality. Patients who had sepsis without pulmonary infection as a cause of admission were at lower risk of mortality (*P* = 0.04).

Multivariate logistic regression analysis showed that the presence of gastrointestinal bleeding, intracranial haemorrhage and septic shock significantly increased the likelihood of dying, whereas causes of ICU admission and APACHE II scores had no influence (Table 5).

## Discussion

We found that the mortality rate was high in SLE patients admitted to the ICU. The most common cause of ICU admission was lung injury/respiratory failure, followed by sepsis/systemic inflammatory response syndrome, cardiogenic causes and neurological disorders. The occurrences of gastrointestinal bleeding, intracranial haemorrhage and septic shock during the ICU stay significantly increased the likelihood of dying.

Recent studies [1-3] have demonstrated a greater reduction in mortality in SLE patients than in the general population over the past few decades. The 10-year survival rate in retrospective series has been 75–85%, with more than 90% of patients surviving longer than 5 years [1-3,16,17]. Nevertheless, outcomes and prognosis in acutely ill SLE patients admitted to the ICU have rarely been investigated. In 1996, Ansell and coworkers [10] reported a retrospective study of SLE patients in the ICUs of two hospitals. They investigated a total of 30 patients and demonstrated high mortality rate in SLE patients in critical care units (47%), similar to the rate in the present study (47%). However, they found that the only pretreatment factor that predicted a poor outcome was the presence of renal involvement due to SLE *per se*. Survival analysis for patients with and those without renal involvement revealed a difference in long-term survival (maximum follow-up period of 120 months) but not in ICU mortality rate. A multivariate analysis of prognostic factors was not performed in that study because of the small number of patients included. We performed a multivariate analysis in 51 SLE patients admitted to the ICU. Although renal involvement due to SLE was not predictive of patient outcome in the ICU, we identified more than one variable influencing mortality rate in our study.

The average ICU mortality from 1992 to 2000 in our hospital was around 29%, which is lower than the mortality rate in SLE patients admitted to the ICU (47%). The other ICU patients might have different clinical characteristics compared with SLE patients. The data show that the SLE patients requiring ICU admission had poorer outcomes than did other critically ill patients admitted to the ICU.

In one study [4], renal damage, thrombocytopenia, lung involvement, SLE Disease Activity Index greater than or equal to 20 at presentation, and age 50 years or older at diagnosis were all predictive of mortality in univariate and multivariate analyses in SLE patients over a 20-year follow-up period. However, the rate of ICU admission in these patients was not mentioned. In the present study these factors were not associated with ICU and in-hospital mortality in SLE patients. The APACHE II score was of little value in predicting outcome, probably because it could not effectively estimate the influence of underlying systemic diseases and the occurrence of possible complications in the SLE patients admitted to the ICU. Gastrointestinal bleeding, intracranial haemorrhage and septic shock during the ICU stay were associated with a greater risk of death, indicating that clinical course and medical care – not the pretreatment morbidity and acute physiological condition – play key roles in influencing the prognosis of SLE patients in the ICU.

The incidence of gastrointestinal haemorrhage in SLE patients is approximately 5% [18]. Previous studies showed that the incidence of gastrointestinal haemorrhage among the general population of patients admitted to the ICU was 3.5–5% [19,20]. In the present study we found that the incidence of gastrointestinal bleeding among SLE patients was much higher (Table 1) than that in the general cohort of patients admitted to the ICU.

We also found intracranial haemorrhage, including brainstem haemorrhage, subarachnoid haemorrhage and frontal lobe haemorrhage, to be a factor that increases the risk of dying. Acute stroke (infarction or intracranial bleeding) in patients admitted to the ICU with non-neurological problems occurred in 1.25% [21]. Subarachnoid haemorrhage occurred in 10 out of 258 patients with SLE in a previous study [22]. Nevertheless, the actual frequency of and factors contributing to intracranial haemorrhage in SLE patients remain undefined. In the ICU it is often difficult to make a diagnosis of cerebrovascular accident in SLE patients with altered mental status, metabolism-induced focal motor abnormalities, or impaired speech because of mechanical ventilation. On the other hand, many factors may contribute to the pathogenesis of acute stroke, including coagulopathy, hypertension, long-term steroid use and lipid disorders. Early diagnosis and appropriate treatment of intracranial haemorrhage are therefore important aspects of intensive care for SLE patients.

We identified various infectious pathogens in SLE patients. The immunocompromised status associated with the disease itself appears to be primarily responsible for the development of infectious complications [23]. Glucocorticoids and immunosuppressive drugs may increase the risk for infections and the number of types of infections that develop. We found the pathogens in SLE patients in the ICU to vary considerably, and the development of septic shock is a major prognostic factor in these patients. In many patients infections develop in the setting of active lupus undergoing aggressive treatment; alternatively, the manifestations of active lupus can mimic infection clinically. It is sometimes difficult to clarify the site of infection and to initiate antimicrobial therapy promptly. Godeau and coworkers [24] found corticosteroid administration to be related to in-hospital mortality in patients with systemic rheumatic disease who were admitted to the ICU. However, that phenomenon did not present in our study. The differences between studies might be due to several factors. First, our study included a relatively small number of patients. Second, a high percentage of patients received steroid treatment before ICU admission and during the ICU stay (92.2% and 80.4%, respectively); more SLE patients not receiving steroid treatment would be necessary to demonstrate a difference between these two groups. However, Godeau and coworkers [24] found corticosteroid treatment to be related to in-hospital mortality, but other immunosuppressive treatments were not related to outcomes in their study. Further large prospective studies might provide more clinical information about the relationship between immunosuppressive agents and outcomes in this patient population.

There some limitations to the present study. Because of the relatively small number of patients included, the patients studied may not be representative the clinical features of the SLE population. Also, because of the retrospective design, the study lacks information on initial disease activity and laboratory data at the first visit to the hospital, although these clinical features may change after medical treatment but before ICU admission. Initial parameters may have little influence on ICU outcomes, but this could not be tested in the present study.

## Conclusion

The mortality rate in critically ill patients with SLE is high. We posit that gastrointestinal bleeding, intracranial haemorrhage and septic shock are significant prognostic factors in SLE patients admitted to the ICU. In contrast, the causes of ICU admission and APACHE II score are not significantly associated with mortality.

## Key messages

• 	The mortality rate in critically ill SLE patients remains high.

• 	We found that gastrointestinal bleeding, intracranial haemorrhage and septic shock were significant prognostic factors in critically ill patients with SLE.

## Abbreviations

APACHE = Acute Physiology and Chronic Health Evaluation; ARDS = acute respiratory distress syndrome; ICU = intensive care unit; SLE = systemic lupus erythematosus.

## Competing interests

The author(s) declare that they have no competing interests.

## Authors' contributions

C-LH participated in study design and drafted the manuscript. K-YC conceived the study, participated in its design and helped to draft the manuscript. P-SY participated in study design and data collection. Y-LH participated in study design and data collection. H-TC participated in study design and data collection. W-YS performed statistical analysis. C-LY participated in study design. P-CY participated in study design.

## Figures and Tables

**Table 1:** Clinical features of patients with systemic lupus erythematosus admitted to the intensive care unit

Clinical feature	Value
Age (years; mean [range])	29 (12–55)
Female (*n* [%])	47 (92.2)
APACHE II score (mean [range])	19 (9–37)
White blood cell count (×10^9^/l; mean [range])	8.0 (2.2–136.0)
Platelet count (×10^9^/l; mean [range])	132.0 (17.0–474.0)
Thrombocytopenia (*n* [%])	23 (45.1)
Neutropenia (*n* [%])	2 (3.9)
Pulmonary manifestations (*n* [%])	
Consolidation	29 (56.9)
Interstitial	19 (37.3)
Pleural effusion	25 (49.0)

APACHE, Acute Physiology and Chronic Health Evaluation.

**Table 2:** Causes of admission to the intensive care unit in critically ill patients with systemic lupus erythematosus

Cause of admission	Total	Noninfectious	Infectious
Cardiogenic	11 (18.3)		
Cardiogenic shock		4 (6.6)	
Ventricular arrhythmia		2 (3.3)	
Cardiogenic pulmonary oedema		2 (3.3)	
Pericardial effusion with cardiac tamponade		3 (5.0)	
Lung injury/respiratory failure	25 (41.6)		
Pneumonia with ARDS (including one pulmonary tuberculosis)			14 (23.3)
Noncardiogenic pulmonary oedema		7 (11.6)	
Interstitial pneumonitis		1 (1.6)	
Pulmonary embolism		1 (1.6)	
Haemothorax		1 (1.6)	
Upper airway obstruction		1 (1.6)	
Sepsis without pulmonary infection	13 (21.7)		
Unknown origin of infection			9 (15.0)
Infective endocarditis			1 (1.6)
Peritonitis			1 (1.6)
Cellulitis			1 (1.6)
Meningoencephalitis (tuberculous)			1 (1.6)
Neurological disorder	11 (18.3)		
Status epilepticus			7 (11.6)
Intracranial haemorrhage on admission			3 (5.0)
Ischaemic stroke			1 (1.6)

Values are expressed as number (%). ARDS, acute respiratory distress syndrome.

**Table 3:** Disease courses and outcomes of patients with systemic lupus erythematosus admitted to the intensive care unit

Courses and outcomes	Number (%)
Need for mechanical ventilation	35 (68.6)
Steroid use in the ICU	41 (80.4)
Total parenteral nutrition	8 (15.6)
Continuous venovenous haemofiltration	11 (21.6)
Peritoneal dialysis	4 (7.8)
Haemodialysis	16 (31.3)
Operation	6 (11.8)
Gastrointestinal bleeding in the ICU	15 (29.4)
Intracranial haemorrhage in the ICU	6 (11.8)
Pneumothorax in the ICU	4 (7.8)
Septic shock in the ICU	15 (29.4)
Length of ICU stay (days; mean [range])	7 (1–68)
Death in the ICU	24 (47.0)
Death in the hospital	24 (47.0)

ICU, intensive care unit.

**Table 4:** Variables that possibly influence the mortality of patients with systemic lupus erythematosus admitted to the intensive care unit: univariate analysis

Variable	*n*	Died (*n* [%])	*P*
APACHE II score			
>19 (median value)	24	11 (45.8)	0.361
≤ 19	27	9 (33.3)	
Previous seizure attack before admission			
Yes	14	3 (21.4)	0.110
No	37	17 (45.9)	
Sepsis without pulmonary infection on admission			
Yes	13	2 (15.4)	0.04
No	38	18 (47.4)	
Abnormal complete blood count			
Yes	41	23 (50.0)	0.005
No	10	0 (0)	
Gastrointestinal bleeding in the ICU			
Yes	15	11 (68.7)	0.01
No	36	13 (29.5)	
Intracranial haemorrhage in the ICU			
Yes	6	5 (83.3)	0.018
No	45	15 (33.3)	
Concurrent septic shock in the ICU			
Yes	15	14 (93.3)	<0.001
No	36	6 (16.7)	

Included are Acute Physiology and Chronic Health Evaluation (APACHE) II score and variables moderately associated (*P* < 0.2) with survival. ICU, intensive care unit

**Table 5:** Variables that significantly influence the mortality of patients with systemic lupus erythematosus admitted to the intensive care unit: multivariate analysis

Variable	Death: RR (95% CI)	*P*
Gastrointestinal bleeding in the ICU		
Yes	6.97 (0.98–49.68)	0.05
No	1	
Intracranial haemorrhage in the ICU		
Yes	18.68 (1.13–307.06)	0.04
No	1	
Concurrent septic shock		
Yes	77.06 (6.85–866.90)	<0.001
No	1	

CI, confidence interval; ICU, intensive care unit; RR, relative risk.
